# Era of Vaccination Heralds a Decline in Incidence of Hepatitis A in High-Risk Groups in China

**DOI:** 10.5812/hepatmon.838

**Published:** 2012-02-29

**Authors:** Zhuang Fangcheng, Wang Xuanyi, Chen Mingding, Jiang Liming, Wu Jie, Jiang Qi, Gong Yuanping, Qian Wen, Xin Yajuan, Mao Jiangsen

**Affiliations:** 1 Institute of Viral Disease, Zhejiang Academy of Medical Sciences, Hangzhou, China; 2 Biomedical Research Institute, Fudan University, Shanghai, China; 3 Center for Disease Control (CDC), Beijing, China; 4 Key Lab for Bio-tech Vaccine Research, Hangzhou, China; 5 Center for Disease Control (CDC), Shenshi, China; 6 Center for Disease Control (CDC), Jiaojiang, China

**Keywords:** Hepatitis A, Vaccination, China

## Abstract

**Background:**

Hepatitis A was ranked first among all of the different types of viral hepatitis in China, which occurred an average of 500,000 cases annually during the 1980’s. A live attenuated hepatitis A vaccine was applied in preventing of the disease in 1992, large scale used in vaccination program in 1995, and incorporated in the Expanded Program of Immunization in 2008 in China.

**Objective:**

The objective of this study was to determine whether, and to what extent, the decline in the incidence of hepatitis A in China was the result of hepatitis A (HA) vaccination.

**Materials and Methods:**

Official documents and longitudinal serological follow-up studies were reviewed to compare the incidence of HA before and after the introduction of the vaccine.

**Results:**

National trends in the incidence of HA in China saw rates decrease by 92.7% in 2009, compared to the levels seen in 1992. A mass vaccination program was carried out in 3-18 year old children (Wuhan City, China), and its protective efficacy was 85.4%. In a mass vaccination program of an entire population (Shenshi County, China), the annual HA incidence decreased from 359.7/100,000 to 17.7/100,000 (almost 20.3 times). There was a significant relationship found between vaccine coverage and the incidence of HA, the correlation of the negative regression was significant at the 1% (Kendall rank correlation, significant level P < 0.05).

**Conclusions:**

In summary, this study highlights the important role of implementing a vaccination program in decreasing the incidence of HA, and the large protective efficacy of such a strategy, as demonstrated in China

## 1. Background

Hepatitis A (HA) is an acute infectious disease caused by the hepatitis A virus (HAV). HA usually occurs in the form of a local epidemic, and it is mainly transmitted via the fecal-oral route. It is estimated that the world-wide incidence of the disease exceeds 1.4 million cases each year, with the health cost being between 1.5 to 3.0 billion dollars annually [1]. HA is also ranked first occurred cases among all of the different types of viral hepatitis in China, especially in the southeast coastal areas. An average of 500,000 HA cases were reported annually to the Ministry of Health (MOH) in China during the 1980s, this figure represents approximately 5.5 million infections per year when anicteric disease and asymptomatic infections are taken into account as well. During a shellfish associated outbreak of HA in 1988, in Shanghai, China, approximately 320,000 cases were reported [2]. In the year 1990, the incidence of HA was 52.58/100,000 in China, and 114.90/100,000 in the Zhejiang Province (located in the southeast region of China). In 1992, a live attenuated hepatitis A vaccine (H2 strain) became available in China for use among children aged 1 year or older. The vaccine was developed and licensed by State Food and Drug Administration (SFDA), China [3][4], and meets the requirements of both SFDA China and the World Health Organization (WHO) for the manufacture of biological substances [5][6]. In 1995, the Advisory Committee on Immunization Practice from the Center for Disease Control (CDC) in China recommended a target of HA vaccination for selected high-risk populations such as; children aged 1 to 15 years, medical practitioners employees in food factories, and residents in endemic regions. In 2008, the Chinese MOH incorporated the vaccine into the Expanded Program of Immunization (EPI) in China, which means that in this program all infants aged 1 year are immunized free of charge. Influenced by this policy, vaccine coverage was well accepted in the target population. The vaccine is now widely included in immunization programs to prevent HA epidemics in China, as well as in a number of other countries [7][8].

## 2. Objectives

The objective of this study was to determine whether the decreasing incidence of HA seen in China was the result of HA vaccination, and if so, to what extent this could be attributed to the program.

## 3. Materials and Methods

The incidence of HA per 100,000 people was determined by using the data from HA cases reported through the National Infectious Disease Surveillance System (NIDSS) under the leadership of the national Chinese Center for Disease Control and Prevention (CDC). A reportable case was defined as an acute illness with the discrete onset of symptoms and jaundice, and/or elevated serum aminotransferase levels in a person who tested positive for the IgM antibody to the HA virus. HA infections are reportable by law from clinics in rural areas and hospitals to the local county CDC in all jurisdictions that report to the NIDSS. The 4th Chinese National Census in 1990 estimated the population to be approximately 1.16 billion, and in the 5th National Census in 2000 it was approximately 1.3 billion. More detailed national level estimates by; age, sex, race, and ethnicity in 1990 and 2000, were obtained from the National Census Bureau, and regional data was obtained from the Regional Census Bureau. Official documents and policy decisions in the field of HA vaccination were reviewed and its use in the EPI were also employed. Some data from the longitudinal serological follow-up studies (9) was used to compare the incidence of HA before and after the introduction of the HA vaccine. Based on these data, the relationship between the annual HA incidence and vaccine coverage was analyzed. Most of the data was collected from Wuhan (a large city in the central region of China), and from Shensi County and Jiaojiang City (located in the east coast areas of the Zhejiang Province, China).

##  4. Results

### 4.1. National Trends in Hepatitis A Incidence in China

Before the 1990s, there were several peaks of HA outbreaks occurring over a period of 8-10 years, the highest peak was in 1989 (768,000 cases, 62.63/100,000), the main contributors to the HA outbreak were infected cases from Shanghai and the eastern provinces of China. Between 1990 to1992 the annual incidence of HA in China was 50/100,000. The live H2 strain vaccine was administrated in 1992 and from that time the incidence of HA gradually decreased. In 2009, the rate of 3.57 per 100,000 (43,841 reported cases) was 92.7% lower than that in 1992 (49.08 per 100,000, among 602,591 reported cases) (Table 1).

**Table 1:** Hepatitis A Incidence in China from 1990 to 2009

	**Annual HA Cases**	**Annual Incidence of HA(1/100,000)^b^**	**Year**	**Annual HA Cases**	**Annual Incidence of HA (1/100,000)b**
1990 a	584.353	47.60	2000 a	134.094	10.92
1991	637.717	51.95	2001	121.532	9.90
1992	602.591	49.08	2002	111.068	9.05
1993	457.895	37.30	2003	99.383	8.10
1994	353.388	28.79	2004	93.587	7.62
1995	254.242	20.71	2005	73.349	5.97
1996	238.331	19.41	2006	68.667	5.59
1997	215.844	17.58	2007	77.135	6.28
1998	183.373	14.94	2008	56.052	4.57
1999	203.177	16.55	2009	43.841	3.57

^a^ Total population in China in 1990: 1.160 billion, No.4 National Population Census; in 2000: 1.295 billion, No.5 National Population Census.

^b^ Annual incidence of HA is calculated from the verified data from the 1990 and 2000 censuses.

### 4.2. Correlation of the National Hepatitis A Incidence With Hepatitis A Vaccine Output

**Figure 1:**
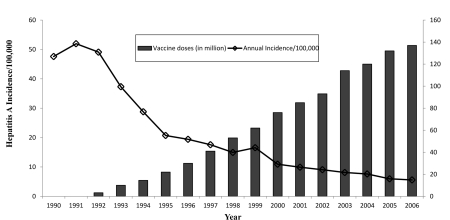
Relationship Between Vaccine Output and HA Incidence (1/100,000) in China from 1990-2006. 1983-1989 before vaccination; 1990-1991 clinical III trial; 1992-2002 mass vaccination program. Kensall RankTest τ_2_ = -0.931, P_2_ < 0.01.

The national incidence rate of HA decreased dramatically from 1992 to 2006. During the same period, the annual cumulative output of a live attenuated (H2 strain) HA vaccine from a major manufacturer, (Pukang Company, located in Hangzhou, China) increased from 2.9 million doses in 1992 to 138.2 million doses in 2006 (165.3 million doses in 2009) (Figure 1).

### 4.3. The Protective Efficacy of Live Attenuated Hepatitis A Vaccine in the Mass Vaccination Program

1. Protective efficacy in Wuhan City by mass vaccination (Table 2)

**Table 2:** HA Vaccine Protective Efficacy in Wuhan City by Mass Vaccination in 1994

	**HA Incidence in December 1993(1/100,000)**	**HA Incidence in December 1994(1/100,000)**	**Protective Efficacy, %**
3-6, y	290.81	49.94	82.82
7-12, y	272.94	19.55	92.84
13-18, y	123.91	31.02	74.97
Total (3-18, y)	229.22	33.50	85.40

2. The mean of the annual HA incidence in the total population of the Shenshi County was 359.7/100,000 before the vaccine was applied (from 1983 to 1989), and a notable decrease to 17.7/100,000 (almost 20.3 times less) took place after vaccination was implemented (from 1990 to 1999). By comparing the number of HA cases before and after vaccination, it is clear that the number of HA cases decreased from several hundred to zero, especially among children from 1 to 15 years old (Table 3).

**Table 3:** The Difference of Hepatitis A Incidence in Shengsi County Before and After Live HA Vaccination

	**Whole Population**			**Children 1-15 Years**		
	**HA Cases**	**HA Incidence (1/100,000)**	**Mean Pop/Annually**	**HA cases**	**HA Incidence (1/100,000)**	
**Before Vaccine Inoculation**						
1983	81780	239	292.25	22004	191	868.02
1984	81622	245	300.16	21078	196	929.84
1985	82310	147	178.59	19907	116	582.71
1986	82750	234	282.78	19458	185	950.77
1987	83320	244	292.85	18745	195	1040.78
1988	83579	789	944.02	16902	563	3330.96
1989	84214	187	222.06	18109	150	828.32
**After Vaccine Inoculation**						
1990 a	84652	42	49.61	18139	14	77.18
1991 a	85209	44	51.64	18279	15	82.29
1992	85603	20	23.36	18446	11	59.63
1993	85850	17	19.80	18473	7	37.89
1994	85890	9	10.48	18450	3	16.26
1995	86207	10	11.60	18837	7	37.16
1996	86314	7	8.11	19030	1	5.25
1997	86041	1	1.16	17285	0	0.00
1998	85550	1	1.17	16631	1	6.01
1999	85264	1	1.17	15131	0	0.00

^a^ Clinical III trial carried out in 1990 and 1991

**Figure 2:**
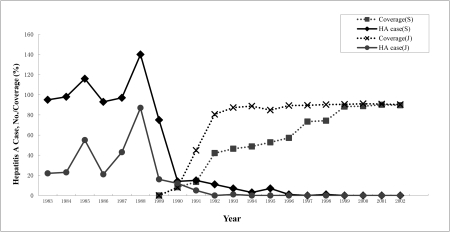
Correlation Between Vaccine Coverage and HA Incidence in 1–5 Year Old Children Group

3. A significant correlation was found between vaccine coverage and HA incidence. Before 1989, there were no HA vaccinations in the Shengsi County and Jiaojiang City. From 1990 to 1991, the H2 strain vaccine clinical phase Ⅲ trial was initiated in these two regions, and a mass vaccination project focusing mainly on 1-15 year old children has been ongoing since 1992. Vaccine coverage has improved annually, and the incidence of HA in these locations has decreased dramatically (Figure 2). During the years 1996 to 1998 vaccine coverage increased rapidly from 57.22% to 74.28%, and only 2 cases were found, neither of which were among people who had been vaccinated. In Jiaojiang City vaccine coverage reached 88.73% in 1994 during the early stages of the project, and remained at a steady level between 84.75% and 91.12% in the following years. So far there has not been any HA cases in this susceptible group at any point in the 9 year period between 1994 and 2002. The correlation of negative regression between vaccine coverage and the HA incidence rate was significant at the 1% level using the Kendall rank correlation test (τ1 = -0.825, P1 < 0.01; τ2 = -0.931, P2 < 0.01). Through the mass vaccination project, the incidence rate of HA in these two regions has dropped dramatically by more than 95% [10].

## 5. Discussion

This current report describes a dramatic decline in the incidence of HA in China. The reported rate of HA was 47.60 per 100,000 in the population in 1990, in contrast, this rate decreased to only 3.57 per 100,000 people in 2009. This decline was more significant in the eastern regions where the vaccination program has had a major impact; on the other hand this level of rate decrease applied less in the western regions. In 2009, the incidence of HA was the lowest level recorded according to all records in 20 years of surveilance, and the provisional 2009 rate of 3.57 reported cases per 100,000 represented yet a further decrease. This decline coincided with the implementation of the EPI programme of HA vaccination for children. It should be noted that the greater decrease in incidence rates of HA in children (especially between 1-15 years) may have resulted in a shift in the age profile of reported cases. This fundamental shift in the epidemiologic patterns of HA has accompanied the decline in decreased rates in all age groups, as is shown in the data [10][11] from both Shensi County and Jiaojiang City.

Simultaneously, along with the vaccination program, peoples’ sanitation conditions have also improved and lifestyle changes may also have contributed to the decrease in the incidence rates, however the vaccination program is considered to have had the major impact [2]. Determining the role of vaccination which has resulted in the observed changes in HA epidemiology is complicated by the historical incidence patterns of HA, and also by improvements in Chinese sanitation conditions and peoples’ lifestyles. Unfortunately, there is no data that allows the various components of this decline to be differentiated, therefore, the proportion of the decrease resulting from improvements in socio-economic status and the proportion of the decline that is attributable to the use of the vaccine cannot be accurately determined [2]. Nevertheless, it can be concluded that the HA vaccine has played a remarkable role in the decreasing incidence of HA.

HA was the main infectious disease in Wuhan City in the central area of China before 1993, and the annual average incidence of HA was 215.14 per 100,000 from 1984 to 1993. A mass vaccination program was carried out by the CDC in Wuhan City in Dec. 1993 with the target population being children aged 3 to 18 years. The average protective efficacy was 85.4% [12]. Shensi County, an island in the east of the Zhejiang Province in China, had a high HA incidence before 1990. Taking its special geographical location into consideration, a mass vaccination was carried out in 1-15 year old children in order to reach the goal of eliminating HA cases from occurring in this area. The first vaccine program that began in March 1990, was named the live HA vaccine clinical III trial. Almost all of the children aged 1-15 years were inoculated with a live vaccine giving coverage of 90%, other people such as those working in the food industry and medical clinics were also inoculated. In the following years, the vaccine effectively prevented the occurrence of HA under the surveillance of the local CDC doctors. Only one HA case was found in the whole population, and that person had not been vaccinated (one HA case occurred in 1997, 1998, and 1999 separately). Since then, there have been no further HA epidemics in the entire county, thanks to the mass vaccination program [9]. Before 1989, HA cases were reported annually, ranging from 75 to 140 (average 102 HA case/year) in Shengsi County, and from 21 to 87 (average 38 HA case/year) in Jiaojiang City. Because there was only an insignificant change of the 1-15 year group population (less than 1,600 changed in 85,264 in whole 1-15 year aged population Shensi County and 84,614 in aged population Jiaojing City), the measurement of HA case/year was used as an indicator in (Figure 2). Comprehensive information on HA coverage could provide a useful context in which to interpret the key findings presented in this report. Unfortunately, only very limited data are available and vaccination coverage among adults is not assessed. However, the available information indicates that HA vaccine coverage in the high risk group (1-15 year old children) in this research region is high. From the trial, it has been revealed that there was a significant negative correlation between vaccine coverage and HA incidence rates [13]. This available information concerning vaccine use, indicates that the observed declines in rates among children appear to have been achieved with a modest level of vaccination coverage, supporting the hypothesis of a strong herd immunity effect.

When vaccine coverage is relatively high (> 80%), the group would be more likely to develop immune herd protection as a result. Additional evidence of such an effect was seen in Shengsi County where the vaccine of children (approximately 42% in 1992 increased to 89% in 1999) resulted in a substantial reduction in disease rates in adults. Similar findings have been reported from other countries in which routine HA vaccination of infants or children has been implemented [14][15]. A recent article that explains the results of modeling the relationship between HA incidence and vaccine coverage also found a strong herd immunity effect, accounting for more than one third of the estimated number of cases prevented by vaccination [16]. The HA incidence rates from 1990 to 1992 were used as baseline rates and therefore represented the pre-vaccination era, because HA incidence had varied almost equally except for the peak in 1998. The baseline rates were compared with those of the most recent years rather than with the average from multiple years, because implementation of the vaccination had occurred in stages in different provinces [17], and was still expanding, thus there was no well-defined post vaccination period. Our analyses used passive surveillance data reported through the NIDSS, which collects information on symptomatic, serological confirmed HA cases, and this has a number of previously described limitations. These reports represent only a portion of all infections, due to the fact that asymptomatic infections and some symptomatic cases are not reported. The incidence HA has historically exhibited a pattern of periodic fluctuation, thus further monitoring in the future is required in order to determine the extent to which the declines that have occurred will be sustained, and can be attributed to vaccination [11][18]. In addition, more data on vaccine coverage levels are needed to better describe the relationship between HA vaccine usage and disease rates.

In summary, implementation of this vaccination strategy revealed a significant decrease in the incidence of HA in children and a large herd immunity effect. All the above mentioned results demonstrate that the H2 strain vaccine can and will play an important role in preventing and controlling future HA epidemics in China.
